# Current concepts and future of noninvasive procedures for diagnosing oral squamous cell carcinoma - a systematic review

**DOI:** 10.1186/s13005-015-0063-z

**Published:** 2015-03-25

**Authors:** Esam Omar

**Affiliations:** grid.412892.40000000417549358Department of Oral and Maxillofacial Surgery, College of Dentistry, Taibah University, Madinah, Saudi Arabia

**Keywords:** Oral cancer, Noninvasive methods, Optical biopsy, Saliva-based diagnosis

## Abstract

**Background:**

Oral squamous cell carcinoma (OSCC) has a remarkably high incidence worldwide, and a fairly serious prognosis, encouraging further research into advanced technologies for noninvasive methods of making early diagnoses, ideally in primary care settings.

**Objectives:**

Our purpose was to examine the validity of using advanced noninvasive technologies in diagnosis of OSCC by identifying and evaluating relevant published reports.

**Data source:**

MEDLINE, EMBASE, and CINAHL were searched to identify clinical trials and other information published between 1990 and 10 June 2014; the searches of MEDLINE and EMBASE were updated to November 2014. Study selection: Studies of noninvasive methods of diagnosing OSCC, including oral brush biopsy, optical biopsy, saliva-based oral cancer diagnosis, and others were included.

**Data extraction:**

Data were abstracted and evaluated in duplicate for possible relevance on two occasions at an interval of 2 months before being included or excluded.

**Data synthesis:**

This study identified 163 studies of noninvasive methods for diagnosing OSCC that met the inclusion criteria. These included six studies of oral brush biopsy, 42 of saliva-based oral diagnosis, and 115 of optical biopsy. Sixty nine of these studies were assessed by the modified version of the QUADAS instrument. Saliva-based oral cancer diagnosis and optical biopsy were found to be promising noninvasive methods for diagnosing OSCC.

**Limitation:**

The strength of evidence was rated low for accuracy outcomes because the studies did not report important details required to assess the risk for bias.

**Conclusions:**

It is clear that screening for and early detection of cancer and pre-cancerous lesions have the potential to reduce the morbidity and mortality of this disease. Advances in technologies for saliva-based oral diagnosis and optical biopsy are promising pathways for the future development of more effective noninvasive methods for diagnosing OSCC that are easy to perform clinically in primary care settings.

## Introduction

Oral cancer is the eighth most common cancer worldwide and represents a significant disease burden. If detected at an early stage, survival from oral cancer is better than 90% at 5 years, whereas survival of patients presenting with late stage disease is only 30%. The 5-year survival rate for oral cancer has remained less than 50% over the last 50 years for the following reasons [1,2]: (i) most oral cancers (60%) are diagnosed at advanced stages (III and IV); and (ii)) oral cancer is subject to the “field cancerisation phenomenon”, having the highest risk of development of second primary tumours of any cancer.

Although the precise sequence and number of events required for tumourigenesis remains unknown, understanding of tumourigenesis may help in development of more effective methods for diagnosis and treatment. A recent series of experiments performed by Hahn et al. [3,4] and Hanahan and Weinberg [3,4] demonstrated that the following six important steps are likely necessary for a cancer to develop [3-5]: (i) acquisition of autonomous proliferative signalling; (ii) inhibition of growth inhibitory signals; (iii) evasion of programmed cell death; (iv) immortalisation; (v) acquisition of a nutrient blood supply (angiogenesis); and (vi) acquisition of the ability to invade tissue.

Accounting for 96% of all oral cancers, squamous cell carcinoma (SCC) is usually preceded by dysplasia presenting as white epithelial lesions on the oral mucosa (leukoplakia). Leukoplakias develop in 1–4% of the population [6]. Malignant transformation, which is quite unpredictable, develops in 1–40% of leukoplakias over 5 years [6]. Dysplastic lesions in the form of erythroplakia (red lesions) carry a 90% risk of malignant conversion [6]. Tumour detection is further complicated by a tendency towards field cancerisation, leading to multicentric lesions, all of which may not be clinically visible [7]. These benign lesions are often biopsied surgically; in most cases multiple follow-up biopsies are indicated. The following disadvantages of surgical biopsies can discourage patients from agreeing to further diagnostic biopsies: (i) fear and stress; (ii) pain and damage to healthy tissue; (iii) risk of infection; (iv) temporary disability and discomfort; and (v) cosmetic concerns.

The peak incidence of oral squamous cell carcinoma (OSCC) is between the ages of 45 and 75 years. The increasing number of older persons worldwide, together with concomitant increases in the incidence of malignancies, are creating a pressure on healthcare systems [8]. The percentage of people aged over 65 years will grow substantially between 2010 and 2030, the predicted annual growth rate being 2.8% (EURON, 2004). Healthcare is expected to become increasingly inadequate over the coming years. If reliance is placed on current conventional diagnostic technologies, which are subjective and depend on examiner experience, provision of sufficient quality and quantity of these would place further demands on the availability of healthcare [8]. There is a thus a strong need to develop new, objective, noninvasive methods for diagnosing OSCC that can be performed by primary care providers: these would improve the outcome of this disease and minimise strain on speciality referral centres. Minimally invasive interventions are critical to improving healthcare efficiency, enhancing the quality of care provided, and reducing cost. The trend is toward facilitating the making of early diagnoses of OSCC by GPs or dentists possible in primary care settings. The major advantages of these techniques comparing with conventional surgical biopsy are [8]: (i) reduced fear and stress; (ii) reduced pain and damage to healthy tissue; (iii) reduced risk of infection; (iv) shorter recovery times and quicker return to work; (v) very ill patients can also be investigated and treated; (vi) more cosmetically pleasing outcomes; and finally (vii) improved cost-effectiveness of diagnostic procedures (polyclinic).

Minimally invasive intervention is considered one of the most important developments in the healthcare industry. The global market for minimally invasive intervention is steadily growing, its annual growth rate being approximately 10% [8]. As patients become more aware of the rapid technological advances, they demand less invasive procedures [8].

In 1986, Bouquot noted that as many as 10% of US adults have some form of oral abnormality that requires histopathological assessment [9]. One of the oldest noninvasive techniques is application of toluidine blue (TB), which has an affinity for nucleic acids and therefore binds to nuclear material in tissues with a high DNA and RNA content [9-11]. However, because it is highly subjective, inexperienced practitioners cannot use this technique to diagnose OSCC. A reliable method for diagnosing oral mucosal abnormalities has been and remains the scalpel biopsy. Because most patients are fearful and stressed about the prospect of scalpel biopsies, oral brush biopsy has been developed as a less invasive substitute [9-11]. For decades, dental healthcare professionals have measured the buffering capacity and bacterial content of saliva to assess the risk of developing tooth decay. Today, scientific and technological advances in biochemistry, microbiology, and immunology are leading to the discovery of new biomarkers in saliva that can be used to detect systemic illnesses such as ischemic heart disease, heart failure, and cancer [12-18]. Saliva diagnosis received a major boost in 2002 when the US National Institute of Dental & Craniofacial Research funded a project under the title of “Development and validation technologies for saliva-based diagnostics”. This project has created a collaborative team of engineers, experts in nanotechnology and biomedical diagnostic fluids, and scientists in oral biology to develop a point-of-care technique that is automated, miniaturised and has a multiplexed platform (lab-on-a-chip) [12,13]. The saliva-based diagnoses are a new noninvasive pathway for diagnosing OSCC in the future with development of point of care.

Optical biopsy involves light–tissue interactions and different types of spectroscopy depending on the nature of the tissues and type of excitation light used [14-18]. The development of optical techniques for noninvasive diagnosis of OSCC is an ongoing challenge in biomedical optics. Optical diagnostics have provided a reliable objective resource that can be used to give instant diagnoses of soft and hard tissue diseases. Multiple studies have suggested that different technologies of optical biopsy are almost as accurate as surgical biopsy. Most of the experimental spectroscopy work in head and neck malignancies has involved fluorescence spectroscopy, Raman spectroscopy, elastic scattering spectroscopy (ESS), optical coherence tomography (OCT), and micro-endoscopy (for the upper aerodigestive tract) [14-18]. Despite significant advances in cancer treatment, early detection of cancer and its curable precursors remains the best way of optimising patient survival and quality of life. The most common noninvasive methods for diagnosing OSCC are summarised in List 1.

**List 1 Summary of the most common non-invasive methods for diagnosing oral squamous cell carcinoma**

- Toluidine blue
- Oral brush biopsy
  - Conventional oral brush biopsy
  - Oral brush biopsy coupled with computer-assisted analysis
- Saliva-Based Oral Cancer Diagnosis
  - Genomic substances
  - Transcriptomc substances
  - Proteomic substances
- Light-based detection systems:
  - Chemiluminescence (ViziLite Plus; Microlux/DL, Orascoptic-DK)
  - Tissue fluorescence imaging (VELscope)
- Optical Biopsy:
  - Tissue fluorescence spectroscopy
  - Raman spectroscopy
  - Elastic scattering Spectroscopy
  - Differential path-length spectroscopy
  - Nuclear magnetic resonance spectroscopy
  - Confocal reflectance microscopy (CRM)
  - Optical Coherence Tomography
  - Angle-resolved low coherence interferometry (A/LCI)
  - Others
- Biomarkers:
  - DNA-analysis
- Laser capture microdissection

## Methods

This systematic review studied noninvasive methods for diagnosing OSCC.

### Key questions

This study was designed to answer the following four key questions.

1. Why is TB staining unreliable?
2. What is the sensitivity and specificity of oral brush biopsy?
3. How does the accuracy of different types of optical biopsy compare with that of surgical biopsy for diagnosis of OSCC?
4. What changes may develop in oral saliva in OSCC patients and those who are at high risk of OSCC and are these changes diagnostic for OSCC?

### Data sources and selection of articles for possible inclusion

Exception for one study from 1986, MEDLINE, EMBASE, the Cochrane Library, and CINAHL were searched to identify clinical trials and other information published between 1990 and 10 June 2014; the searches of MEDLINE and EMBASE were updated to November 2014. The major terms and concepts searched included (but were not limited to) the following: surgical biopsy, optical biopsy, saliva-based oral cancer diagnosis, and saliva changes in people with cancer. Table 1 provides a complete list of search terms and strategies.

**Table 1:** **A complete list of search terms and strategies**

**Set**	**Concept**	**Search statement**
1	Oral	Oral cavity
2	Oral biopsy	Surgical biopsy, Toluidine blue staining, Oral brush biopsy, Optical Biopsy
3	Optical Biopsy	Fluorescence spectroscopy, Raman spectroscopy, Elastic scattering spectroscopy, Differential path-length spectroscopy, Optical Tomography
4	Fluorescence spectroscopy	Auto-fluorescence spectroscopy, Enhanced dye fluorescence, Ratio imaging
5	Oral diseases	Tumors, carcinoma
6	Combined set	2, 5
7	Combined set	3, 5
8	Combined set	3, 4, 5
9	Saliva	Saliva-based oral cancer diagnosis,
10	Oral Saliva changes in cancer patients	Genomic substance, Transcriptomic mRNA, Proteomic substances
11	Combined set	9, 10
12	Combined set	6, 7, 8, 11
13	Limit by publication type	5,6, 8, 11, 12: Not letter or editorial or news or comments or case report or notes or conference paper
14	Diagnostics filter	13 and (predictive value of tests or sensitivity and specificity or accuracy or diagnostic accuracy or precision or likelihood) or (false or true) or (positive or negative)
15	Clinical trials filter	14 and (Randomized controlled trials or random allocation or double-blind method or single-blind method or cross-over studies or crossover procedure or double blind procedure or single blind procedure or crossover design or double-blind studies or single-blind studies or triple-blind studies or random assignment or exp controlled study/or exp clinical trial/or exp comparative study or intermethod comparison or parallel design or control group or prospective study or case control study or major clinical study) or Case control studies/or Cohort/or Longitudinal studies/or Evaluation studies/or Prospective studies
16	Combined set	14, 15
17	Patient satisfaction	13 and (patient satisfaction or pain measurement or pain assessment or visual analog scale or quality of life).

The fundamental a priori criteria for inclusion of studies were that they involved direct comparison of a noninvasive method with surgical biopsy; 10 or more patients were enrolled for the purpose of making a primary diagnosis of an oral mucosal abnormality; and the findings were published as an English-language, full-length, peer-reviewed article. List 2 shows all of the study inclusion criteria.

**List 2 Inclusion criteria**

1. Studies addressing Key Questions 1 and/or 2
2. Studies prospective in design
3. Enrollment of 10 or more patients for the purpose of diagnosis
4. The patients assessed by the gold standard (surgical biopsy)
5. Non-invasive methods in diagnosis of OSCC
  Toluidine blue staining,
  Oral brush biopsy,
  Optical Biopsy
6. Optical Biopsy:
  Fluorescence spectroscopy,
  Raman spectroscopy,
  Elastic scattering spectroscopy,
  Differential path-length spectroscopy,
  Optical Tomography
  Nuclear magnetic resonance spectroscopy
7. Auto-fluorescence spectroscopy,
  Enhanced dye fluorescence,
  Ratio imaging
8. Saliva:
  Saliva-based oral cancer diagnosis
9. Oral Saliva changes in cancer patients:
  Genomic substance,
  Trans-criptomic mRNA,
  Proteomic substances
10. English-language
11. Full-length article
12. Peer-reviewed article

**Figure 1:**
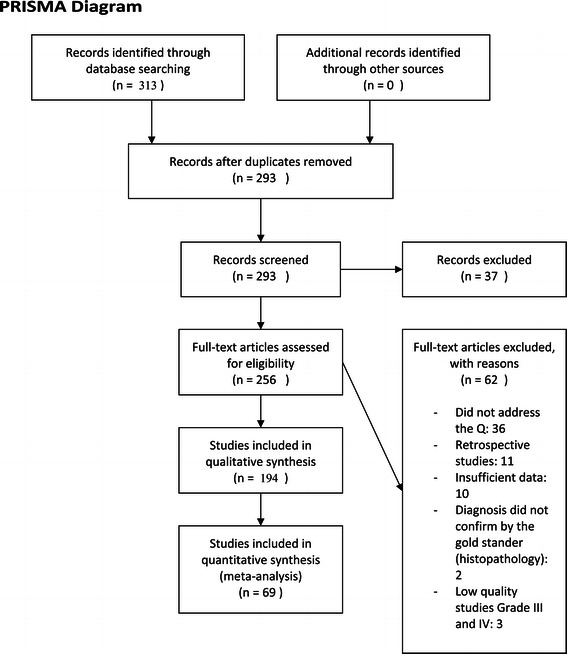
**PRISMA diagram.**

Abstracts of articles identified by the investigator were evaluated in duplicate for possible relevance on two occasions at intervals of 2 months; 313 abstracts were thus identified. When exclusion and inclusion criteria were applied at the abstract level, 62 abstracts were excluded. The 286 full-length articles of studies that seemed relevant at the abstract level were then obtained and the full articles examined to ascertain whether they met the inclusion criteria; 194 of these articles met the inclusion criteria (Figure 1).

### Data abstraction and quality assessment

Standardised data abstraction forms were created, the relevant data abstracted from each article, and the accuracy of the abstracted data verified. A modified version of the Quality Assessment of Diagnostic Accuracy Studies (QUADAS) instrument developed by Whiting et al. was used to assess the internal validity of each of the included studies because we considered the original form of QUADAS adequate (List 3) [19]. The modifications made to this instrument included the following: (i) were the patients assessed by the gold standard? (surgical biopsy); (ii) were the patients assessed by a reference standard regardless of the biopsy results?; (iii) was funding for this study provided by a source with no obvious financial interest in the findings?; (iv) was the design prospective?; (v) were complete data reported?; (vi) were all patients assessed by the reference standard?; (vii) were interreader differences accounted for?; and (viii) were readers and outcome assessors blinded? List 4 provides the full list of items modified.

**List 3 (Modified Diagnostic Accuracy Studies (QUADAS) instrument developed by Whiting et al*.*)**

- **Category 1: Spectrum composition**
  - Was the spectrum of patients described in the paper and was it chosen adequately?
  - Were selection criteria described clearly?
  - Was the method of population recruitment consecutive?
  - Was the setting of the study relevant?
  - Was disease prevalence and severity reported? (not included in QUADAS)
- **Category 2a: Index test and reference standard: Selection and execution**
  - In light of current technology, was the reference standard chosen appropriate to verify test results?
  - Is it possible that a change in the technology of the index test has occurred since this paper was published? (not included in QUADAS)
  - Was there an abnormally long time period between the performance of the test under evaluation and the confirmation of the diagnosis with the reference standard?
  - Was the execution of the index test described in sufficient detail to permit replication of the test?
  - Was the execution of the reference standard described in sufficient detail to permit replication of the test?
  - Did the whole sample, or a random selection of the sample, receive verification using a reference standard of diagnosis?
  - Did all patients receive the same reference standard regardless of the index test result?
  - Were the results of the index test incorporated in the results of the reference standard?
  - Was the cut-off value pre-specified or acceptable in light of previous research? (not included in QUADAS)
  - Was treatment started based on the knowledge of the index test results before the reference standard was applied? (not included in QUADAS)
  - Category 2b: Index test and reference standard: Interpretation
  - Were the index test results interpreted blind to the results of the reference standard?
  - Were the reference standard results interpreted blind to the results of the index test?
  - Was clinical data available when test results were interpreted?
  - Is data presented on observer or instrument variation that could have affected the estimates of test performance? (not included in QUADAS)
- **Category 3: Analysis**
  - Were appropriate results presented (sensitivity, specificity, likelihood ratios, diagnostic odds ratios, predictive values) and were these calculated appropriately? (not included in QUADAS)
  - Was a measure of precision of the results presented (confidence intervals, standard errors)? (not included in QUADAS)
  - Were uninterpretable/indeterminate/ intermediate results reported and included in the results?
  - Was the threshold value specified retrospectively based on analysis of the results? (not included in QUADAS)
  - Were reasons for drop-out from the study reported?
  - Were subgroup analyses pre-specified and clinically relevant? (not included in QUADAS)
  - Were results presented in a 2 × 2 data table? (not included in QUADAS)
  - Was any indication of the utility of the test provided? (not included in QUADAS)
- **Category 4: Research Planning**
  - Was an appropriate sample size calculation performed and were sufficient patients included in the study? (not included in QUADAS)
  - Were study objectives clearly reported? (not included in QUADAS)
  - Was there any evidence that a study protocol had been developed before the study was started? (not included in QUADAS)

**List 4 Quality assessment instrument (the full list of items modified)**

1. Was the study prospective in design?
2. Were the patients assessed by the gold standard (surgical biopsy)
3. Were patients assessed by a reference standard regardless of the biopsy results
4. Was funding for this study provided by a source that doesn’t have an obvious financial interest in the findings of the study
5. Did the study account for inter-reader/scorer differences?
6. Were the reader(s) of the investigated procedure blinded to the results of the reference standard?
7. Were readers of the reference standard blinded to the results of the study?
8. Were the readers of the investigated procedure blinded to all other clinical information?
9. Were readers of the reference standard blinded to all other clinical information?
10. The experience of the investigation’s team in the relevant field

The strength of evidence supporting each major conclusion was graded as high, moderate, low, or insufficient. According to the modified version of the QUADAS instrument, the proportion of tools covering each item were classified as follows: Grade I, 75–100%; Grade II, 50–74%; Grade III, 25–49%; and Grade IV, 0–24% [19]. Studies classified as Grades III and IV were excluded; thus, only prospective studies have been were included in this assessment.

### Statistical analysis

Statistical analysis was performed using Microsoft office XL version 2007, which was used to calculate the 30 variables in the modified version of QUADAS [19]. The items were scored by using the terms used in QUADAS, namely, Yes, No, and Unclear. The “Yes” answer was given two points, “No” no points, and “Unclear” one point. The total number of points was divided by six to obtain a score over 10.

## Results

### Evidence of validity of non-invasive methods in diagnosing OSCC

This study identified 163 studies of noninvasive methods for diagnosing OSCC that met the inclusion criteria. These included six studies of oral brush biopsy, 42 of saliva-based oral diagnosis, and 115 of optical biopsy. Sixty nine of these studies were assessed by the modified version of the QUADAS instrument [19] (Table 2).

**Table 2:** **Analysis of studies addressing key questions 1, 2, 3 or 4 that met the inclusion criteria**

**Study**	**Study or biopsy type(s)**	**Quality score**	**Type of study**	**Care setting**	**Country conducted in**	**Funded by**	**Number of patients enrolled**
Epstein et al. 1997 [10]	Toluidine blue	7	Prospective	Department of Dentistry, British Columbia Cancer Agency	Canada	British Columbia Cancer Agency	-
Bouquot et al. 1986 [11]	Toluidine blue	6.5	Prospective	-	USA	-	23,616
Martin et al. 1998 [23]	Toluidine blue	6.3	-	Department of Oral and Facial Surgery, Sunderland Royal Hospital	UK	-	-
Scheifele et al. 2004 [24]	OralCDx® technique	6.4	Prospective	Department of Oral Surgery and Dental Radiology, Zentrum für Zahnmedizin, Campus Virchow, Charité--Universitätsmedizin Berlin	Germany	-	103
Sciubba 1999 [26]	OralCDx® technique	7.3	Prospective	Department of Dental Medicine, Long Island Jewish Medical Center	USA	U.S. Collaborative OralCDx® Study Group	945
Gupta et al. 2007 [26]	Oral brush biopsy	5.8	Prospective	Department of Pathology, Moti Lal Nehru Medical College, Allahabad University	India	-	96
Poate et al. 2004 [28]	Oral brush biopsy	7.5	Prospective	Oral Medicine, Division of Maxillofacial Diagnostic, Medical and Surgical Sciences, Eastman Dental Institute for Oral Health Care Sciences	UK	-	112
Weigum et al. 2010 [29]	Nanobiochip, exfoliative cytology	7.7	Prospective	Department of Dental Diagnostic Science, University of Texas Health Science Center	USA	National Institute for Dental and Craniofacial Research	52
Jokerst et al. 2010 [31]	Nanobiochip	6.6	Prospective	Stanford University School of Medicine	USA	-	-
Wei et al. 2009 [32]	Saliva biomarkers	7.5	Prospective	University of California, Los Angeles School of Dentistry and Dental Research Institute	USA	NIH/National Institute of Dental and Craniofacial Research	-
Floriano et al. 2009 [33]	Saliva-based nanobiochip tests	7.6	Prospective	Department of Chemistry and Biochemistry, University of Texas	USA	National Institute of Dental and Craniofacial Research	41
Liu et al. 2009 [34]	Saliva biomarkers	7.5	Prospective	Department of Mechanical Engineering and Applied Mechanics,	USA	University of Pennsylvania Institute for Translational Medicine and Therapeutics	-
Zimmermann et al. 2007 [36]	Saliva biomarkers	7.8	Prospective	School of Dentistry and Dental Research Institute, University of California	USA	National Institute of Health	-
Xie et al. 2008 [37]	Saliva biomarkers	7	Prospective	Department of Biochemistry, Molecular Biology, and Biophysics, School of Dentistry, University of Minnesota	USA	-	-
Sugimoto et al. 2010 [39]	Saliva biomarkers	8	Prospective	UCLA Medical Center	USA	National Institute of Health	215
Hu et al. 2008 [41]	Saliva biomarkers	7.6	Prospective	Oral Biology and Medicine Division and Dental Research Institute, School of Dentistry, University of California	USA	U.S. Public Health Service	64
Rosin et al. 2000 [44]	Biomarkers; genetic	7.7	Prospective	British Columbia Cancer Agency/Cancer Research Centre	Canada	National Cancer Institute of Canada, Canadian Cancer Society	116
Boyle et al. 1993 [48]	Biomarkers; genetic	7.6	Prospective	Department of Oral Surgery, Johns Hopkins University	USA	-	102
Rosas et al. 2001 [50]	Biomarkers; genetic	7.9	Prospective	Department of Otolaryngology, Head and Neck Surgery, Johns Hopkins University School of Medicine	USA	National Institute of Dental and Craniofacial Research (NIH)	30
Chien et al. 1990 [52]	Biomarkers; genetic	7.5	Prospective	Department of Obstetrics and Gynaecology, First Affiliated Hospital, Human Medical University	China	-	92
Handschel et al. 2007 [54]	Biomarkers; genetic	6.9	Prospective	Department for Cranio- and Maxillofacial Surgery, Heinrich-Heine-University	Germany	-	-
Hasselmann et al. 2001 [55]	Saliva biomarkers; clinical chemistry	6.4	Prospective	Department of Dermatology, Saarland University Hospital	Germany	-	-
Ratajczak et al. 2006 [56]	Biomarkers; cellular	7.2	Prospective	James Graham Brown Cancer Center, University of Louisville	USA	Stem Cell Biology Program	-
García et al. 2008 [57]	Biomarkers; genetic	7.5	Prospective	Department of Medical Oncology, Hospital Universitario Puerta de Hierro	Spain	Ministerio de Educación y Ciencia and the Fundación de Investigación Médica Mutua Madrileña	-
Skog et al. 2008 [59]	Biomarkers; genetic	7.5	Prospective	Department of Neurology, Massachusetts General Hospital, and Neuroscience Program, Harvard Medical School	USA	Wenner-Gren Foundation Stiftelsen Olle Engkvist Byggmästare, Brain Tumor Society, and American Brain Tumor Association	-
Shpitzer et al. 2009 [63]	Saliva biomarkers	7.8	Prospective	Department of Otorhinolaryngology, Rabin Medical Center, Petah Tiqva and Sackler Faculty of Medicine	-	-	19
Vairaktaris et al. 2008 [64]	Saliva biomarkers	7.5	Prospective	Department of Oral and Maxillofacial Surgery, University of Athens Medical School, Attikon Hospital	Greece	-	152
St John et al. 2004 [64]	Saliva biomarkers	7.7	Prospective	School of Medicine, UCLA	USA	National Institutes of Health, UCLA Jonsson Cancer Center	32
Rhodus et al. 2005 [66]	Saliva biomarkers	7.6	Prospective	Department of Oral Medicine, University of Minnesota	USA	-	13
Arellano-Garcia et al. 2008 [67]	Saliva biomarkers	7.5	Prospective	School of Dentistry, Oral Biology and Medicine Division, Dental Research Institute, University of California	USA	U.S. Public Health Service	19
Betz et al. 2002 [111]	Optical biopsy	7.9	Prospective	Department of Oto-Rhino-Laryngology/Head and Neck Surgery, Ludwig Maximilians University	Germany	Wilhelm Sander Foundation	85
Leunig et al. 2000 [112]	Optical biopsy	7.5	Prospective	Department of Otorhinolaryngology--Head and Neck Surgery, University of Munich	Germany	Wilhelm Sander Foundation	8
Betz et al. 1999 [101]	Optical biopsy	7.7	Prospective	Department of Oto-Rhino-Laryngology/Head & Neck Surgery, Ludwig Maximilian University	Germany	Wilhelm Sander Foundation	49
Kulbersh et al. 2007 [103]	Optical biopsy	7.5	Prospective	Department of Surgery, Division of Otolaryngology-Head and Neck Surgery, University of Alabama	USA	American Cancer Society, University of Alabama at Birmingham Comprehensive Cancer Center Core Grant NIH	33 Models
Ebenezar et al. 2012 [113]	Optical biopsy	7.3	Prospective	Anna University, Department of Medical Physics	India	-	25
Duraipandian et al. 2012 [118]	Optical biopsy	7.8	Prospective	National University of Singapore, Department of Bioengineering, Faculty of Engineering, Optical Bioimaging Laboratory	Singapore	National University of Singapore	2748
Guze et al. 2014 [133]	Optical biopsy	7.8	Prospective	Department of Oral Medicine, Infection and Immunity, Harvard School of Dental Medicine, Divisions of Oral Medicine, Dana-Farber Cancer Institute and Brigham and Women’s Hospital	USA	-	18
Krishnakumar et al. 2013 [125]	Optical biopsy	7.5	Prospective	Department of Physics, Annamalai University	India	-	-
Sahu et al. 2013 [126]	Optical biopsy	7	-	Chilakapati lab, ACTREC, Tata Memorial Centre	India	Advanced Centre for Treatment Research and Education in Cancer	70
Singh et al. 2013 [128]	Optical biopsy	7.3	Prospective	Chilakapati lab, ACTREC, Tata Memorial Centre	India	Advanced Centre for Treatment Research and Education in Cancer	84
Singh et al. 2012 [127]	Optical biopsy	7.5	Prospective	Advanced Centre for Treatment Research and Education in Cancer, Chilakapati Laboratory	India	Advanced Centre for Treatment Research and Education in Cancer	104 subjects
Deshmukh et al. 2011 [129]	Optical biopsy	7	Prospective	Chilakapati Laboratory	India	Advanced Center for Treatment Research and Education in Cancer	10
Oliveira et al. 2006 [130]	Optical biopsy	7.2	Prospective	Grupo de Optica Biomédica, Instituto de Pesquisa e Desenvolvimento, Universidade do Vale do Paraíba	Brazil	-	123 spectra
Malini et al. 2006 [131]	Optical biopsy	7.5	Prospective	Center for Laser Spectroscopy, Manipal Academy of Higher Education	India	Government of India	216 spectra
Krishna et al. 2004 [132]	Optical biopsy	6.8	Prospective	Center for Laser Spectroscopy, Manipal Academy of Higher Education	India	Government of India	-
Jerjes et al. 2004 [135]	Optical biopsy	7.5	Prospective	Department of Oral and Maxillofacial Surgery, University College London Hospitals	UK	Eastman Dental Institute, UCL, UCLH Head and Neck Centre	13
Sharwani et al. 2006 [140]	Optical biopsy	7.6	Prospective	Oral and Maxillofacial Surgery, Eastman Dental Institute	UK	Eastman Dental Institute, UCL, UCLH Head and Neck Centre, London, UK	25
Mourant et al. 2000 [137]	Optical biopsy	7	Prospective	Los Alamos National Laboratory, Bioscience Division	USA	-	-
Mourant et al. 1998 [138]	Optical biopsy	7.2	Prospective	Chemical Sciences and Technology Division, Los Alamos National Laboratory	USA	-	-
Lovat et al. 2006 [139]	Optical biopsy	7.8	Prospective	National Medical Laser Centre, Department of Surgery, Royal Free and University College Medical School, University College	UK	National Cancer Institute	181
A’Amar et al. 2013 [144]	Optical biopsy	7.5	Prospective	Department of Biomedical Engineering, Boston University	USA	-	42
Denkçeken et al. 2013 [145]	Optical biopsy	6.9	Prospective	Biomedical Optics Research Unit, Department of Biophysics, Faculty of Medicine, Akdeniz University	Turkey	Biomedical Optics Research Unit	10
Qi et al. 2012 [143]	Optical biopsy	7.7	Prospective	Department of Surgery and Cancer, Imperial College London	UK	ERC grant (China Scholarship Council)	-
Upile et al. 2012 [142]	Optical biopsy	7.8	Prospective	Head & Neck Unit, University College London Hospitals	UK	Eastman Dental Institute, UCL, UCLH Head and Neck Centre	73
Canpolat et al. 2012 [141]	Optical biopsy	7.6	Prospective	Department of Biophysics, School of Medicine, Akdeniz University	Turkey	-	28
Lau et al. 2009 [146]	Optical biopsy	7.9	Prospective	Massachusetts Institute of Technology, George R. Harrison Spectroscopy Laboratory	USA	National Institutes of Health	-
Müller et al. 2003 [136]	Optical biopsy	7.9	Prospective	Massachusetts Institute of Technology	USA	National Institutes of Health	91 tissue sites from 15 patients
Amelink et al. 2004 [147]	Optical biopsy	7.5	Prospective	Department of Radiation Oncology, Erasmus Medical Centre	The Netherlands	Dutch Technology Foundation STW, Applied Science Division of NWO and the Technology Program of the Ministry of Economic Affairs	-
Sterenborg et al. 2009 [148]	Study type(s) or biopsy	7.6	Prospective	Center for Optical Diagnostics and Therapy, Erasmus Medical Centre	The Netherlands	Dutch Technology Foundation STW, Applied Science Division of NWO and the Technology Program of the Ministry of Economic Affairs	21
de Visscher et al. 2012 [150]	Study type(s) or biopsy	7.5	Prospective	University Medical Centre Groningen, Department of Oral and Maxillofacial Surgery, Division of Oncology	The Netherlands	Dutch Technology Foundation STW, Applied Science Division of NWO and the Technology Program of the Ministry of Economic Affairs	54 male Wistar rats
Amelink et al. 2011 [149]		7.7	Prospective	Centre for Optical Diagnostics and Therapy, Department of Radiation Oncology, Erasmus Medical Centre	The Netherlands	Dutch Technology Foundation STW, Applied Science Division of NWO and the Technology Program of the Ministry of Economic Affairs	18
Karakullukcu et al. 2011 [151]	Study type(s) or biopsy	7.6	Prospective	The Netherlands Cancer Institute, Antoni van Leeuwenhoek Hospital, Department of Head and Neck Oncology and Surgery	The Netherlands	Dutch Technology Foundation STW, Applied Science Division of NWO and the Technology Program of the Ministry of Economic Affairs	-
Kanick et al. 2008 [152]	Study type(s) or biopsy	7.5	Prospective	Erasmus Medical Centre, Department of Radiation Oncology, Centre for Optical Diagnostics and Therapy	The Netherlands	Dutch Technology Foundation STW, Applied Science Division of NWO and the Technology Program of the Ministry of Economic Affairs	-
Adalsteinsson et al. 1998 [174]	Study type(s) or biopsy	7.5	Prospective	Department of Radiology, Stanford University	USA	-	12
El-Sayed et al. 2002 [175]	Study type(s) or biopsy	7.8	Prospective	Cancer Care Manitoba	Canada	Cancer Care Manitoba	135
Maheshwari et al. 2000 [176]	Study type(s) or biopsy	7.6	Prospective	Department of Radiology, University of North Carolina	USA	University of North Carolina	37
Mukherji et al. 1997 [177]	Study type(s) or biopsy	7.8	Prospective	Department of Radiology, University of North Carolina School of Medicine	USA	-	49
Kunkel et al. 2003 [178]	Biochemical	7.8	Prospective	Department of Oral and Maxillofacial Surgery, University Hospital Mainz	Germany	-	118

### Selected important study quality items

**Figure 2:**
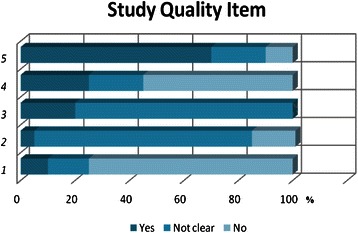
**Study quality measures.**

These 5 study quality measures have been judged as highly important for reducing the risk for bias when addressing the key questions of this review. “Reported sufficient relevant clinical information” refers to whether the study reported clear and sufficient information about the study design, patient inclusion and exclusion criteria and characteristics and biopsy methods to fully address the key questions and fully assess the potential for bias in the study design. “Index test results blinded” refers to whether readers were aware of the reference standard of biopsy results. “Differential verification bias avoided” refers to whether the reference standard was chosen regardless of the biopsy results. “Representative spectrum enrolled” refers to whether the patient’s group enrolled in the study resembles the usual patient population seen in clinical practice. “Avoided selection bias” refers to whether all or consecutive patients enrolled in the study were clearly selected by applying consistent inclusion and exclusion criteria (Figure 2).

#### TB staining

Vital staining is the staining of living cells or tissues. The earliest technique, developed by Paul Ehrlich in 1885, involved immersion of freshly removed tissue in methylated blue. There are two techniques for vital staining, namely, intravital staining within the living body (in vivo) and supravital staining outside the body, which usually involves preparation of slides of detached cells [20,21]. TB is a basic thiazine metachromatic dye with high affinity for acidic tissue components, thereby staining tissues rich in DNA and RNA. TB staining is a simple, inexpensive and sensitive tool for identifying early OSCC and high-grade dysplasias [22]. A 1% aqueous TB solution is applied for 30 seconds, this acidophilic metachromatic nuclear stain helps to differentiate areas of carcinoma in situ or invasive carcinoma from normal tissue. TB staining is highly sensitive and moderately specific for malignant lesions. It has less sensitivity for premalignant lesions, up to 58% false negatives having been reported for identifying mild-to-moderate dysplasia [22,23]. Rosenberg and Cretin stated that the sensitivity of TB staining in oral cancer screening ranges from 93.5% to 97.8%, and the specificity from 73.3% to 92.9% [20].

#### Oral brush biopsy

The goal of the highly sensitive and specific technique of oral brush biopsy is to provide a sample by a less painful and simpler means than scalpel or punch biopsy. The accuracy of brush tests has been the subject of many published studies. In every study in which oral lesions have been simultaneously assessed by both a brush biopsy and surgical biopsy, this test has been shown to have both sensitivity and specificity well over 90% [24,25]. Oral brush biopsy uses a circular bristled brush that has been designed to access and sample all epithelial layers, including the basal cell layer and the most superficial aspects of the lamina propria [25]. Brush biopsy has many advantages: it is a chair-side, easy to perform, painless test that can be used to evaluate any suspicious lesion, including common small white and red oral lesions, and to rule out dysplasia. Gupta et al. combined conventional oral brush biopsy with the application of TB to identify suspicious mucosal areas [26]. Scully et al. stated that the sensitivity of brush biopsy in detection of dysplasia or OSCC is 71.4%, whereas the specificity is only 32% [27]. Oral brush biopsy coupled with computer-assisted analysis has been developed as a technique for evaluating unexplained clinically detectable alterations of the surface epithelium of the oral mucosa; where cancer or pre-cancer is suspected, the sensitivity is up to 40%. [25]. This technique is based on quantitative cytomorphometry and DNA aneuploidy with computer-assisted analysis [25]. However, the limited specificity of current cytology-based analysis is still a major impediment to early oral cancer detection and intervention [24-28]. Given that exfoliative cytology also gathers cellular DNA, RNA, and protein biomarkers, new diagnostic techniques targeting early tumour biomarkers and molecular transformation could enhance the role and utility of oral cytology in clinical diagnostics. Exfoliative cytology based on a nanobiochip sensor platform for oral cancer detection was recently described in a pilot study examining both molecular and morphological biomarkers associated with oral dysplasia and malignancy [29]. In this study, oral epithelial cells were captured on a membrane filter with pores smaller than the cell size followed by immunofluorescent labelling for the well-known epidermal growth factor receptor biomarker. Concurrently, the cytoplasm and nuclei were stained with the fluorescent dyes phalloidin and 4′,6-diamidino-2-phenylindole, respectively, for cytomorphometric measurements. The nuclear area, nuclear diameter, nuclear/cytoplasmic ratio, and EGFR expression in malignant and dysplastic oral lesions were found to differ significantly from those in normal control epithelial cells. This technique reportedly has 97–100% sensitivity and 86% specificity [24,28,29].

#### Saliva-based oral cancer diagnosis

Saliva can be considered a mirror of bodily health. The multifarious components of saliva not only protect the integrity of oral tissues, but also provide clues to various local and systemic conditions and diseases. The components of saliva are continually being explored as markers of various diseases and for monitoring general health [30]. In the past few years, multiplex biomarker detection systems have emerged through remarkable progress in the development of lab-on-a-chip and point-of-care technologies [31]. The goal of these efforts is to use developments in nano/micro-electrical–mechanical technology to build automated, miniaturised, and multiplexed platforms for rapid assays and readouts. In general, the principles of conventional enzyme-linked immunosorbent assay and/or nucleic acid hybridisation are applied, often via either electrochemical sensors [32] or microbead reactors [33,34]. The electrochemical approach uses gold electrode arrays (multiplex chips) in which one set of electrodes (working, counter and reference electrodes) is applied, with a cyclic square wave electrical field to facilitate chemical reaction, for one analyte measurement, followed by amperometric readout [32].

Tumour cells may inhibit or produce biochemical substances referred to as tumour markers. These can be normal endogenous products that are produced at a greater rate in cancer cells or the products of newly switched on genes that are quiescent in normal cells [33-35]. Tumour markers may be present as intracellular substances in tissues or as released substances in circulating body fluids such as serum, urine, cerebrospinal fluid, and saliva. Until recently, tumour markers were analysed in fluids other than saliva, such as cerebrospinal fluid, blood, and urine. However, with recent technological advances in diagnostic techniques, the role of saliva as a tool for diagnosis has grown exponentially. Saliva-based oral cancer diagnosis is a noninvasive alternative to serum testing and has an overall accuracy rate of about 85%. It is an effective modality for diagnosis, determining prognosis of oral cancer and monitoring post-therapy status [33-35].

Relevant markers include oncogenes (e.g., C-myc, c-Fos, C-Jun), anti-oncogenes (e.g., p53, p16), cytokines (e.g., transforming growth factor-β1, interleukin (IL)-8, and -1β), growth factors (e.g., vascular endothelial growth factor, epidermal growth factor, and insulin-like growth factor*),* extracellular matrix-degrading proteinases (MMP1, MMP2, MMP9), hypoxia markers (hypoxia-inducible factor-α, carbonic anhydrase-9), epithelial-mesenchymal transition markers (e.g., E-cadherin, N-cadherin, and β-catenin), epithelial tumour factors (Cyfra 21–1), cytokeratins (CK13, 14 and 16), microRNA molecules, and hypermethylation of cancer-related genes (p16 and death-associated protein kinase) [36-43].

#### Genomic substances

Markers in the form of changes in the host DNA of dysplastic or cancer cells include point mutation, deletion, translocation, amplification, and methylation (Table 3). Loss of heterozygosity in chromosomes 3p, 9q, 13q, and 17p is considered an early event in oral carcinogenesis. In their study, Rosin et al*.* [30,44] found that allelic loss at 3p and 9q increases the risk of malignant transformation by 3.8-fold; the risk further increases to 33-fold when loss of heterozygosity occurs in chromosomes 4q, 8p, 11q, 13q and 17p in addition to the former. Mitochondrial DNA mutations have also been useful for detecting exfoliated OSCC cells in saliva. Such mutations have been identified in 46% of patients with head and neck cancer [45], and have been identified by direct sequencing in 67% of saliva samples from OSCC patients [45,46]. The p53 gene, which is located on chromosome 17p13.1, exhibits mutation in 50–70% of epithelial tumours [47,48]. Loss of heterozygosity of the p53 allele has been reported in 22% of cases of pre-cancer and 20% of oral cancer. Other genes related to p53 and the cell cycle, such as p16, p27, p63, and p73 have been found to be altered to varying degrees in oral cancer [47,48]. Using plaque hybridisation, Boyle et al. [48] identified tumour-specific p53 mutations in 71% of saliva samples from patients with head and neck cancer. Cyclin-dependent kinase inhibitor 2A, which is involved in the retinoblastoma pathway of the cell cycle, appears to be methylated in 23–67% of primary OSCCs. CDH1 gene is responsible for cell adhesion, promotes metastasis when mutated, and shows promoter methylation in up to 85% of tumours [49,50]. Rosas et al*.* [50] identified aberrant methylation of at least one of the genes p16, O6-methylguanine-DNA methyltransferase, or death-associated protein kinase in OSCC and detected promoter hypermethylation in 65% of matched saliva samples from OSCC patients. Amplification and over-expression of c-MYCIN-MYC has been observed in 20–40% of oral cancers. Das et al*.* [51] have reported amplification of 11q13, which contains 1NT2, HST1, and cyclin D oncogenes, in 30–50% of patients with oral cancer. The specificity and positive predictive value were higher for saliva than for serum (88.0% vs. 59.8% and 54.2% vs. 28.8%, respectively). In the case of OSCC, many studies have noted a significant increase in salivary concentrations of Cyfra 21–1, tissue polypeptide-specific antigen, and cancer antigen 125 with a sensitivity of 71%, specificity 75%, negative value 71%, and positive predictive value 75%. On the other hand, carcinoembryonic antigen and cancer antigen19-9 are not detected with statistically significant frequency [51-54].

**Table 3:** **Genomic substance**

**Genome**	**Functions**	**Type of abnormality**	**Reported rate in the saliva**
Mitochondrial DNA		mutations	67%
p53 gene: Tumor-suppressor genes	Cell-cycle regulation Senescence, cell-cycle progression	mutations	71%
p16* : Tumor-suppressor genes	Cell-cycle regulation Senescence, cell-cycle progression	Hypermethylation	47%
DAP-K*	kinase whose expression is required for IFN-γ-induced apoptosis	Hypermethylation	33%
MGMT*		Hypermethylation	23%
CDKN2A	Control of cell cycle, arrest cell cycle at G1& G2act like a Tumor-suppressor genes	Hypermethylation	30.2%
CDH1	Encodes Ca++ dependent cell to cell adhesions	Hypermethylation	-
c-MYCIN: Proto-oncogenes	Cell growth, apoptosis	amplification	20-40%
Cyclin D oncogenes: Proto-oncogenes	Cell-cycle regulation	amplification	87%

*p16, MGMT,DAP-K (methylation of at least one of these genes in 65%).

#### Transcriptomic mRNA

It has been speculated that salivary mRNA is contained in apoptotic bodies [55,56] or actively released in exosomes or microvesicles [57,58]. Researchers [59,60] have compared the clinical accuracy of salivary versus blood RNA biomarkers for oral cancer detection and found four RNA biomarkers that have a sensitivity and specificity of 91% and 71%, respectively, and a collective receiver operator characteristic value of 0.95 (Table 4). A study by Speight and Morgan found seven mRNA molecules to occur significantly more frequently in OSCC patients than in healthy controls [61]. These included the following: (i) IL-8, [44,61]; (ii) IL-1β, which takes part in signal transduction, proliferation, inflammation, and apoptosis [44,61]; (iii) dual specificity phosphatase 1, which has a role in protein modification, signal transduction, and oxidative stress [44,61]; (iv) H3 histone, family 3A, which has DNA binding activity [44,61]; (v) ornithine decarboxylase antizyme 1, which plays a part in polyamine biosynthesis [44,61]; (vi) S100 calcium binding protein P, which has a role in protein binding and calcium ion binding [44,61]; and (vii) spermidine/spermine N1-acetyltransferase, which takes part in enzyme and transferase activity [44,61].

**Table 4:** **Trascriptomic RNA**

**Biomarker**	**Gene functions**	**Mean fold increase**	**Sensitivity (%)**	**Specificity (%)**
*DUSP1*	Protein modification; signal transduction; oxidative stress	2.60	59	75
*H3F3A*	DNA binding activity	5.61	53	81
*IL1B*	Signal transduction; proliferation; inflammation; apoptosis	5.48	63	72
*IL8*	Angiogenesis; replication; calcium-mediated signaling pathway; cell adhesion; chemotaxis; cell cycle arrest; immune response	24.3	88	81
*OAZ1*	Polyamine biosynthesis	2.82	100	38
*S100P*	Protein binding; calcium ion binding	4.88	72	63
*SAT*	Enzyme, transferase activity	2.98	81	56

#### Proteomic substances

Carbonylation signifies oxidative damage to proteins: there is reportedly a substantial increase in salivary carbonyls (246%) in OSCC patients, indicating that their epithelial cells are being exposed to significant free radical attack [62]. The sensitivity and specificity for carbonyls are 90% and 80%, respectively. MMP-9 polymorphism has been shown to be strongly associated with increased risk of developing OSCC [62-64]. Shpitzer et al*.* [63] reported a 39% increase in MMP-9 with a sensitivity of 100% and specificity of 79% in OSCC patients. St John et al*.* [65] and Rajkumar et al*.* [66] found significantly increased concentrations of IL-6 and IL-8 in saliva of OSCC patients (Table 5). Another study reported that patients with OSCC have significantly higher concentrations of IL-8 in saliva than patients with dysplastic oral lesions and normal controls, suggesting its diagnostic value as a marker of malignant transformation of oral premalignant lesions [67]. Arellano-Garcia et al*.* [68] used Luminex xMAP (Austin, TX, USA) technology to show that both IL-8 and IL-1β are expressed significantly more strongly in OSCC patients.

**Table 5:** **Proteomic substances**

**Parameter**	**% Of change**	**Sensitivity (%)**	**Specificity (%)**
Matrix metalloproreinases-9 (MMP-9)	39	100	79
Salivary Carbonyls	246	90	80
8-oxoguanine DNA glycosylase (OGG1)	-16	77	75
phospho-Src	-24	77	75
Ki67	127	58	67
Maspin	-29	100	100
lactate dehydrogenase (LDH)	86	79	42
CycD1	87	100	100

### Light-based systems

Light-based systems depend on the assumption that absorption and reflection of light differs between normal tissues and those with metabolic or structural changes. Vizilite Plus with TBlue system (Zila Pharmaceuticals, Phoenix, AR, USA), LED Dental (White Rock, BC, Canada) Microlux/DL (AdDent, Danbury, CT, USA) and Orascoptic DK (Orascoptic, Middleton, WI, USA) are light-based oral cancer screening aids that have been developed with the aim of assisting identification of early stage precancerous and cancerous lesions. LED Dental, a handheld device with an illuminated chemiluminescent stick, emits visible light in the 430 nm wavelength that causes fluorescent excitation of certain compounds in tissues. After the patients have rinsed their mouths with acetic acid, the oral cavity is examined. With Microlux (AdDent) and ViziLite (Zila Pharmaceuticals), the oral cavity is examined with a battery-powered fibreoptic visible light source rather than a chemiluminescent stick; again, prior rinsing with acetic acid is required. These devices are not sensitive or specific for diagnosing any type of oral lesion. Only pathologic examination of tissue can definitively determine the biologic nature of a lesion [68-70]. The ViziLite system offers an alternative to white light illumination for visual examination; a disposable chemiluminescent light source illuminates tissue with blue light. Providers observe the reflected blue light to detect abnormal changes in the oral cavity. Initial studies conducted by Epstein et al. [69] and Kerr et al. [70] indicated that the ViziLite potentially aids detection of oral premalignant lesions by improving brightness and sharpness. Epstein et al. used conventional white light and ViziLite illumination to examine 134 patients with oral lesions [69] and reported that two lesions were clinically visible only under ViziLite illumination. Kerr et al. used conventional white light followed by ViziLite illumination to examine 501 patients with histories of tobacco use [70] and reported that six lesions not detected by conventional examination were identified by ViziLite examination. However, other studies in which examinations with ViziLite were performed after conventional oral examinations have reported that ViziLite did not aid in the identification of oral lesions [71-73] in 40 patients in a high prevalence population [71] or in 55 patients referred for assessment of white oral lesions [72,73]. Because assessment by reflectance visualisation and illumination with chemiluminescent light sources is largely subjective and dependent on the experience of the examiner, these are considered inappropriate tools for primary care settings [74].

### Optical biopsy

#### Autofluorescence imaging

It has already been established that autofluorescence optical biopsy can produce diagnostically useful information about human oral cavity tissues [75,76]. Both fluorescence imaging and fluorescence spectroscopy have been used with encouraging results [76]. Tissue autofluorescence has the potential to provide information about biochemical, functional and structural transformations of fluorescent bio-molecular complexes in vivo and has therefore been used to investigate the molecular properties of cells and tissue. Given that pathological transformation, therapeutic interventions [77-90] and developmental changes [88-91] cause biological changes in affected tissues, fluorescence has been increasingly explored as a tool for tissue diagnosis and detection of malignant transformation. Moreover, advances in light delivery and collection systems (fibreoptics) have facilitated the development of fluorescence-based techniques for non- or minimally-invasive, remote investigation of tissues using appropriate endoscopic or catheter systems [92]. Fluorescence is an adaptable means of achieving optical molecular contrast using diverse instruments including spectrophotometers, microarrays, microscopes, and endoscopes. Fluorescence measurements can provide information not only on the specific molecular makeup of a sample but also on the local environment of the fluorescence molecule or fluorophore. Distinct species of fluorophores have been characterised based on their excitation and emission spectra, quantum efficiency, polarisation and fluorescence lifetime [78-86]. Common endogenous fluorophores that are used to characterise tissue include aromatic amino acids (tyrosine, tryptophan, and phenylalanine), structural proteins (elastin, collagens, and collagen cross-links), enzyme metabolic co-factors (nicotinamide adenine [phosphate] dinucleotide [NAD{P}H] and flavin adenine dinucleotide [FAD]), lipid components, and porphyrins [77-90]; commercial systems are now available for measuring autofluorescence in tissues. For example, Xillix Technologies (Richmond, BC, Canada) (now Novadaq Technologies, Bonita Springs, FL, USA) [93], Storz [94], Pentax (Tokyo, Japan) [95] and Richard Wolf (Vernon Hills, IL, USA) [96] have commercialised endoscopic systems such as LIFE, D-light, SAFE-100, and DAFE. These systems are equipped with fluorescence excitation-collection modules and have been designed to analyse autofluorescence contrast to diagnose cancers in the bronchi and gastrointestinal tract. Medispectra (Lexington, MA, USA) and SpectRx (currently Guided Therapeutics, Norcross, GA, USA) [97] have developed devices incorporating fluorescence and reflectance spectroscopy for diagnosis of cancer of the cervix. Also, relatively recently the Food and Drug Administration (FDA) has approved a new device VELscope (LED Dental [98]) for direct visualisation of autofluorescence in the oral cavity and diagnosis of oral carcinoma. In a study of 122 oral mucosa biopsies from 20 patients, Poh et al. have shown that VELscope imaging can identify oral neoplasia in the operating room setting with a sensitivity of 97% and specificity of 94% [99].

#### Fluorescence spectroscopy

It is well known that all tissues fluoresce and that malignant tissues fluoresce less than normal tissues: thus, these tissues have different spectral characteristics. Studies have shown that when an ultraviolet or near ultraviolet light source is used, normal oral mucosa emits more green fluorescence than neoplastic lesions [100,101]. Malignant tissues differ from normal tissues in various physical and chemical characteristics that are altered by subcellular architectural changes such as nuclear grade, nuclear to cytoplasm ratio, mitochondrial size and density, amount of keratin, and elastin to collagen ratio. Several mathematical methods have been proposed for evaluating recorded spectral features of fluorochromes and correlating these with disease states as a form of diagnostic optical biopsy [100,101].

#### Autofluorescence spectroscopy

Autofluorescence imaging has recently been shown to improve detection of premalignant and malignant oral lesions [100,101]. This method is based on absorption of ultraviolet and visible spectrum light by tissue fluorophore molecules (NAD and hydrogen flavin adenine dinucleotide [100,101] [FADH] in the epithelial layer and collagen and elastin in the stroma), which leads to emission of lower energy photons that can be detected as fluorescence from the oral mucosa. Optical fibres may be introduced into tissues through a hollow needle and the tissue signals interpreted by spectrometers [100,101]. Betz et al. compared autofluorescence imaging and spectroscopy of normal and malignant mucosa in 49 patients with head and neck cancer. In 13 of these patients (43.3%), it was subjectively easier to distinguish tumours from their surroundings by observing reduction in green autofluorescence [102]. Spectral analysis showed contrasts in autofluorescence intensities between tumour and normal tissues in 34 patients (94.4%) [102]. Mayinger et al. studied endoscopic detection of oesophageal cancer by autofluorescence spectroscopy. They obtained 129 endogenous fluorescence spectra from normal mucosa and malignant lesions in nine patients with SCC and four with adenocarcinoma of the oesophagus with a sensitivity of 97% and specificity of 95% for diagnosis of oesophageal carcinoma [103].

#### Enhanced dye fluorescence

Fluorescence can be slightly enhanced by exogenously applying fluorescent drugs such as 5-aminolevulinic acid (5-ALA), which induces protoporphyrin IX (PPIX; an important precursor to biologically essential prosthetic groups such as heme, cytochrome c, and chlorophylls) [103-107]. Recent advances include the possibility of extracting true spectra of single fluorophores (chemical compounds that can re-emit light upon light excitation) by mathematically eliminating the undesired influences of scattering and absorption. In addition, it will soon be possible to precisely target tumour-specific enzymes with fluorescent markers (“smart probes”), which will improve both sensitivity and specificity [103-105]. A study of fluorescence imaging with topical application of 5-ALA as a mouth rinse was undertaken at the University College London Hospitals (UCLH) Head and Neck Centre in 71 patients who presented with clinically suspicious oral leukoplakia. A sensitivity of 83–90% and specificity of 79–89% were obtained for differentiation between normal and dysplastic lesions [108]. Several studies at the MD Anderson Cancer Center, Houston, Texas, reported different spectra from normal, dysplastic, and malignant oral mucosa [107-112]. A University Hospital Groningen study reported autofluorescence spectra from 96 volunteers with no clinically observable oral lesions. Skin colour strongly affected autofluorescence intensity, sex differences were found in blood absorption, and alcohol consumption was associated with porphyrin-like peaks. However, all differences apart from those associated with skin colour were of the same order of magnitude as standard deviations within categories [111]. Betz et al. [112] compared normal inspection, combined fluorescence diagnosis and its two main components, autofluorescence and 5-ALA-induced PPIX fluorescence. Biopsy-controlled fluorescence imaging and spectral analysis were performed on 85 patients with suspected or histologically proven oral carcinoma both before and after topical administration of 5-ALA. In terms of tumour localisation and delimitation properties, combined fluorescence diagnosis was clearly favourable over either normal inspection or the two components of combined fluorescence diagnosis. The performance of combined fluorescence diagnosis was hindered by tumour keratinisation but independent of tumour staging, grading, and localisation. In spectral analysis, cancerous tissue showed significantly greater PPIX fluorescence intensity and less autofluorescence intensity than normal mucosa. In this study, the reported sensitivity of enhanced dye spectroscopy was 100% and specificity 51% [112,113]. Leunig et al. studied 58 patients with suspected cancer of the oral cavity by measuring emission spectra of 5-ALA-induced PPIX fluorescence and reported a specificity of 60% and sensitivity of 99% after pathologic evaluation of biopsy specimens [114]. Ebenezar et al. reported that a diagnostic algorithm based on discriminant function scores obtained by fluorescence excitation spectroscopy distinguished well-differentiated SCC from normal lesions with a sensitivity of 100% and specificity of 100% [115].

#### Ratio imaging

This technique compares a photochemical or end metabolic product that is known to be increased in disease states with another product that is known to be depleted. For example, as described above, 5-ALA enhances PPIX, which fluoresces red after excitation with blue light. The same excitation results in green fluorescence of molecules such as NAD and FADH, which are depleted in malignant tissues with a high metabolic rate [105-114]. Shin et al. have reported that the sensitivity of fluorescence imaging techniques ranges from 60 to 97% and their specificity from 75 to 99% [116].

#### Raman spectroscopy

The Raman effect was first discovered by Professor Raman of Calcutta University, for which he was awarded the Nobel prize in 1930 [117]. This effect is based on light’s interaction with matter; when photons are directed towards target matter, most pass through unchanged. However, some photons come into contact with molecules in the matter. Most of these photons interact with the molecules of the substance, exciting them to a partial quantum state, which causes emission of photons at the same frequency as the incident photon [118]. This process is known as elastic scattering. A smaller number of these (approximately 1 in 10^6^ to 1 in 10^8^) photons undergo a process called Raman or inelastic scattering, in which photons are discharged from the material or ‘scattered’ at a differing wavelength than the incident photon; it is this wavelength shift that is recorded in Raman spectroscopy [117,118].

The Raman effect occurs when light impinges on a molecule and interacts with the electron cloud and bonds of that molecule. In the spontaneous Raman effect, which is a form of light scattering, a photon excites a molecule from the ground state to a virtual energy state. When the molecule relaxes, it emits a photon and goes into a different rotational or vibrational state. The difference in energy between the original state and this new state leads to a shift in the emitted photon’s frequency away from the excitation wavelength [94-96,109,110]. A laser-based spectroscopic technique for observing vibrational, rotational, and other low-frequency modes in a system has been developed, enabling characterisation of chemicals and the structure of molecules in a sample. With this technique, laser light interacts with molecular vibrations, phonons or other excitations in the system, resulting in the energy of the laser photons being shifted up or down. These shifts in energy give information about the vibrational modes in the system. This technique delivers a vibrational spectroscopic picture of tissue content, thus providing immediate real-time histology [105-107].

Raman spectroscopy is being investigated as a diagnostic tool for characterising cancer cells and early malignant changes and distinguishing these cells from normal cells. It has a distinct advantage over other optical techniques: it provides information on molecular composition and structure of living tissue [105-107,119,120]. However, significant problems associated with using Raman applications are that signals produced by the Raman effect are inherently weak and Raman bands generally overlap because of various biological constituents, making it difficult to identify individual components correctly. The strong fluorescent background produced by biomedical samples can completely obscure the true Raman signals. In 2000, Raman spectroscopy was used for detection of laryngeal malignancy [121]. Stone and colleagues examined 15 ex-vivo biopsy specimens that had been obtained from patients of varying ages (18 to 79 years). The specimens were allocated to one of three categories (normal, dysplastic, and SCC). Their results demonstrated sensitivities of between 76 and 92%, depending on the tissue type examined, and specificities of over 90% [121]. In a similar study in 2205, Lau et al. examined 47 laryngeal specimens (a mixture of normal, papilloma, and carcinoma) by Raman spectroscopy [122], each spectrum having a five second acquisition time. Sensitivities were similar to those of the study by Stone et al. (69 to 89%), with specificities ranging from 86% to 94%. The authors determined that the ability to discriminate between the tissue types was attributable to spectral differences in the DNA, amino acids, collagen and glycolipids. Lau et al. used Raman spectroscopy to classify tissue obtained from the post-nasal spaces of six cancer and six non-cancer patients [123]. An advantage of utilising Raman spectroscopy in the nasopharynx is the ability to detect submucosal tumours associated with this cancer, preventing the need for random biopsy. Although the study was small, differences were noted in the regions of the spectra associated with collagen, proteins, and lipids. Gniadecka et al. used Raman spectroscopy coupled with neural network analysis to identify skin lesions [124]. Raman spectra were obtained from 22 samples of melanoma, 41 of pigmented nevi, 48 of basal cell carcinoma, 23 of seborrheic keratoses, and 89 of normal skin. These researchers were able to discriminate malignant melanoma from other disorders and normal skin based on the amide I protein region (1660 cm-1) of the spectra with a sensitivity of 85% and specificity of 89%. In 2012, Duraipandian et al. used Raman spectroscopy to obtain 2748 in vivo gastric tissue spectra (2465 normal and 283 cancer). Based on the randomly resampled training database (80% for learning and 20% for testing), they achieved a diagnostic accuracy of 85.6% (95% confidence interval [CI]: 82.9%–88.2%), sensitivity of 80.5% (95% CI: 71.4%–89.6%) and specificity of 86.2% (95% CI: 83.6%–88.7%)] for detecting gastric cancer [120].

In 2009, Harris et al. [125,126] examined 40 patients with Raman spectroscopy, 20 with an established diagnosis of head and neck carcinoma (not all SCC), and 20 aged-matched controls with respiratory ailments. Using a trained genetic algorithm they reported a 75% sensitivity and 75% specificity for each cohort. When mixed samples were used to train the algorithm, they achieved the expected 50% sensitivity and specificity, providing further evidence that the algorithm was able to discriminate between cancer and non-cancer. The oral cavity is readily accessible in a clinic setting and would be ideal for the development of a Raman probe for cancer detection. Many recent studies have been used Raman spectroscopy for diagnosis of OSCC with reported sensitivity of 85% and specificity of 86% [120]. Raman spectroscopy has shown efficacy in differentiation between normal, premalignant, and malignant tissues and can even detect early changes such as cancer-field-effects/malignancy-associated-changes [127-129]. Thus, oral premalignant conditions can be objectively discriminated by Raman spectroscopy [130,131]. However, the need for a dedicated instrument and stringent laboratory conditions limits wide screening applications of this method [127-129]. In 2006, Oliveira et al. reported 100% sensitivity and 55% specificity for near-infrared Raman spectroscopy for oral carcinoma diagnosis [132]. Spectral profiles of normal, malignant, premalignant, and inflammatory conditions reportedly differ markedly [133-135]. Malignancy-induced biochemical changes can radically change spectra from the epithelial region change. Major differences between normal and malignant spectra seem to arise from changes in protein composition and conformation/structure, and possibly from increased protein content in malignant epithelia [134,135]. Guze et al. reported 100% sensitivity and 77% specificity for differentiating premalignant and malignant oral lesions from normal mucosa and benign lesions in humans by Raman spectroscopy [136].

#### Elastic scattering spectroscopy

ESS makes diagnoses by objective statistical and analytical methods rather than by subjective interpretation of images. It provides optical geometrical information that is based on white light reflectance. In ESS, photons hit tissue and are backscattered without changes in wavelength. The relative intensity of this backscattering is influenced by the composition of the interrogated tissue, specifically the relative concentration of scatterers (e.g., nuclei, mitochondria, connective tissue) and absorbers (e.g., haemoglobin). A scattering event carries with it all the characteristics of the cellular components, which are called “scattering centres”. Pathological scattering centres may originate from disorganised epithelial orientation and architecture, changes in morphology of epithelial surface thickness and texture, cell crowding, increased distance from subepithelial collagen layer, enlargement and hyperchromicity of cell nucleus, increased concentration of metabolic organelles, and presence of abnormal protein packages or particles [106,107,136]. The ESS method senses micromorphology changes at the level of subcellular architectural changes, such as nuclear grade, nuclear to cytoplasmic ratio, mitochondrial size and density, without actually imaging the microscopic structure. Because ESS detects changes at a subcellular level, it supplies information that may not be provided by conventional histology. Thus, ESS provides an optical signature of a tumour that greatly depends on that tumour’s morphology [135-138]. The ESS system covers a range of 300–900 nm (light emitted by cellular and subcellular organelles ranges from 330 nm to 850 nm) and uses a pulsed xenon arc lamp as the light source. The system has two fibreoptic probes, one for transmitting light into the tissue and the other for collecting scattered light. The tip of the collecting probe is placed in direct contact with the lesion and a background measurement taken; the lamp is then activated. Next, an ESS measurement is taken within 100 ms with the pulsed lamp, after which the background spectrum is subtracted from the ESS spectrum. The entire measurement processing display takes less than 1 second [104-106]. In summary, ESS provides a point measurement that uses appropriate optical geometry and is sensitive to the size and packing of dense subcellular components (such as the nucleus, nucleolus, and mitochondria) as well as absorption by haemoglobin [139-142].

Müller et al. used three spectroscopic techniques to assess 91 tissue sites from 15 patients with varying degrees of oral malignancy (normal, dysplastic, and cancerous sites) and eight healthy volunteers. By fitting a linear combination of collagen and the reduced form of NADH fluorescence spectra to intrinsic tissue fluorescence spectra that had been excited by 337 nm and 358 nm laser light, these researchers obtained direct biochemical information regarding oral tissue native fluorophores with autofluorescence spectroscopy. They measured wavelength-dependent absorption and scattering coefficients by diffuse reflectance spectroscopy to provide information regarding tissue absorption and structure, such as haemoglobin concentration and stroma density. They then obtained light ESS information resulting from single backscattering from epithelial cell nuclei by subtracting the diffusely reflected component from the measured reflectance; this provided information concerning the size distribution of cell nuclei. They described this method as trimodal spectroscopy and reported a sensitivity and specificity of 96% and 96%, respectively, in distinguishing cancerous/dysplastic (mild, moderate, and severe) from normal tissue. In addition, the authors were able to distinguish dysplastic from cancerous tissue with a sensitivity of 64% and a specificity of 90% [139].

Lovat et al. studied ESS measurements collected in vivo and matched them with pathologic findings of histological specimens taken from identical sites within Barrett oesophagus. They examined 181 matched biopsy sites from 81 patients. ESS detected high risk sites with 92% sensitivity and 60% specificity and differentiated high risk sites from inflammation with sensitivity and specificity of 79% [142]. Sharwani et al. compared findings of ESS with histopathology of oral tissues to ascertain whether this technique could be used as an adjunct or alternative to histopathology for identifying dysplasia. Twenty-five oral sites from 25 patients who presented with oral leukoplakia were examined by ESS using a pulsed xenon-arc lamp and surgical biopsies acquired from each of the examination sites. The results of the acquired spectra were then compared with histopathology. Two sets of spectra were obtained and linear discriminant analysis showed a sensitivity of 72% and a specificity of 75% [143]. Canpolat et al. used elastic light single-scattering spectroscopy to diagnose and demarcate skin malignancy. They performed measurements on 28 lesions in 23 patients and reported that this technique discriminated between malignant and benign lesions with a sensitivity and specificity of 87% and 85%, respectively. Sensitivity and specificity of the system for detecting positive surgical margins on 14 excised biopsy samples were 80% and 90%, respectively [144]. Upile et al. concluded that ESS is a promising means of distinguishing between normal, benign, and malignant skin conditions [145]. In 2012, Qi et al. created multispectral imaging in a rigid endoscope based on ESS [146]. A’Amar et al. used ESS in diagnosis of prostate cancer; the reported sensitivity was 83% and specificity 87% [147]. ESS has also been used in diagnosis of cervical precancerous lesion with a reported sensitivity of 87.5% [148].

#### Differential path-length spectroscopy

Differential path-length spectroscopy, a recently developed fibreoptic point measurement technique, measures scattered photons that have travelled in predetermined path lengths. Differential path-length spectroscopy is considered to be a form of ESS that has fixed photon path length, fixed photon visitation depth, and absolute measurement of absorbers. This technology was developed at the Erasmus Medical Centre, Rotterdam, the Netherlands [149,150]. The system uses a fibre-based diffuse reflection spectrometer with a tungsten-halogen lamp as a white light source. The first spectrometer uses a bifurcated fibre for illumination and collection. A second fibre carries diffusely reflected light to a second spectrometer. The wavelength scales of the spectra recorded by each spectrometer are slightly different. Subtraction of the two measurements selects superficially scattered light [150]. This spectrum is analysed mathematically and translated into a set of parameters that are related to the microvasculature and intracellular morphology. The signals give information about cell biochemistry, intracellular morphology and microvascular properties such as oxygen saturation and average vessel diameter. The reported sensitivity is 69% and specificity 85% [151]. Amelink et al. used differential path-length spectroscopy to study 76 spectra (45 nondysplastic and 31 dysplastic) collected from 27 leukoplakias. Based on a combination of the three variables of blood oxygenation, vessel diameter, and blood volume fraction, nondysplastic and dysplastic leukoplakias can be discriminated with a sensitivity and specificity of 91% and 80%, respectively [152].

#### Optical tomography

This technology uses light scattering either to construct an image, as in OCT, or to measure the average size of different cell structures, thus providing objective information about degree of dysplasia, as in angle-resolved low coherence interferometry (a/LCI).

#### Optical coherence tomography

This is analogous to ultrasound imaging except that it uses light rather than sound. The high spatial resolution of OCT enables noninvasive in vivo “optical biopsy” and provides immediate and localised diagnostic information. The recent development of a Fourier domain mode lock swept source-based OCT system has helped to simultaneously achieve a high speed (>100 kHz A-scan rate) and good spatial resolution (<4 μm). In addition, the development of various miniature scanning probes that provide high-speed three-dimensional OCT pictures has been reported [104-106,153-161]. Wilders-Smith et al. used OCT to image suspicious oral lesions in 50 patients. After imaging, standard biopsy and histopathology were performed. Two investigators who were blinded to OCT and histopathology subsequently diagnosed the lesions. For detecting carcinoma in situ or SCC versus non-cancer, sensitivity was 93% and specificity was 93%; for detecting SCC versus all other pathologies, sensitivity was 93% and specificity was 97% [162,163]. Jerjes et al. compared findings of OCT with histopathological diagnoses of suspicious oral lesions to assess the feasibility of using OCT to identify malignant tissue. Thirty-four oral lesions from 27 patients were assessed with swept-source frequency-domain OCT. Four variables were assessed (changes in keratin, epithelial, and subepithelial layers, and identification of the basement membrane). These researchers confirmed the feasibility of using OCT to identify architectural changes in malignant tissues [164]. Olivo et al. reported strong agreement between OCT-based and histopathological diagnoses with sensitivity and specificity around 93% to 97%, respectively [165]. Volgger et al. evaluated the capability of OCT to differentiate premalignant and early malignant lesions of the upper aerodigestive tract. In an unblinded evaluation, noninvasive and invasive lesions were distinguished with a sensitivity of 88.9% and specificity of 89.0% whereas blinded evaluations led had sensitivities of 100%, 66.7%, and 77.8% and specificities of 75.8%, 71.4%, and 70.3% [166]. In 2014, Pande et al. studied the automated classification of OCT images for the diagnosis of oral malignancy in the hamster cheek pouch and reported the sensitivity and specificity of distinguishing malignant lesions from benign lesions were 90.2% and 76.3%, respectively [167]. They thus demonstrated the feasibility of using quantitative image analysis algorithms to extract morphological features from OCT images to perform automated diagnoses of oral malignancies in a hamster cheek pouch model [167]. The incorporation of OCT in specific tools, like handheld and catheter-based probes, will further improve the implementation of this technology in daily clinical practice [168,169].

#### Angle-resolved low coherence interferometry

A/LCI, an emerging biomedical imaging technology that uses the properties of scattered light to measure the average size of different cell structures, including cell nuclei, directly measures diagnostically relevant subcellular features of epithelial tissues up to 500 μm below the surface. Unlike OCT, which is a subjective method because it requires image interpretation, a/LCI performs an objective analysis of tissue and delivers direct confirmation of precancerous disease to the physician [104-106]. Wax et al. compared the average diameter and texture of cell nuclei in rat oesophagus epithelial tissue with grading criteria established in a previous a/LCI study to prospectively grade neoplastic progression. Overall, the combined studies showed 91% sensitivity and 97% specificity for detecting dysplasia, using histopathology as the standard [170]. Chalut et al. reported that the a/LCI technique distinguishes normal from diseased tissue with a sensitivity of 78% (7/9) and a specificity of 91% (10/11) [171]. Terry et al. evaluated the ability of a/LCI to identify dysplasia by studying tissues from 27 patients undergoing partial colonic resection surgery. They reported that a/LCI was able to separate dysplastic from healthy tissues with a sensitivity of 92.9% (13/14), a specificity of 83.6% (56/67), and an overall accuracy of 85.2% (69/81) [172]. Zhu et al. studied 46 patients with Barrett oesophagus and reported that a/LCI was able to detect dysplasia with 100% sensitivity and 84% specificity [173]. Wax et al. have developed a novel spectroscopic technique for diagnosing disease at the cellular level based on using low-coherence interferometry to detect the angular distribution of scattered light. A/LCI combines the ability of low-coherence interferometry to isolate scattering from subsurface tissue layers with the ability of light scattering spectroscopy to obtain structural information on subwavelength scales. The technology shows promise as a clinical tool for in situ detection of dysplastic or precancerous tissue [174]. Wax et al. and Terry et al. reported sensitivity of this technology in diagnosis of oesophageal lesions of 100% and specificity 85% [175,176].

### Nuclear magnetic resonance spectroscopy (NMR)

NMR exploits the magnetic properties of certain atomic nuclei to determine the physical and chemical properties of atoms or the molecules in which they are contained. It relies on the phenomenon of nuclear magnetic resonance and can provide detailed information about the structure, dynamics, reaction state, and chemical environment of molecules. This technology allows three-dimensional study of atoms in molecules; the larger the magnet, the more sensitive the device. Using NMR, it is possible to view how protein links with DNA [104-106]. NMR has been used to identify metabolic signatures of OSCC compared with normal tissues [177,178]. Clinical studies have confirmed that the choline/creatine ratio is significantly higher in OSCC than in normal tissue [179,180]. An NMR study of ex vivo tumour tissue found higher concentrations of taurine, choline, glutamic acid, lactic acid, and lipids in SCC than in normal tissue [181]. In addition, overexpression of glucose transporters, especially of glucose transporter 1, which is associated with increased glycolytic metabolism, has been reported in OSCC [178]. Other authors who have examined the role of advanced glycated end-products and increased numbers of their receptors in patients with primary gingival carcinoma have shown that expression of these receptors correlates closely with the invasiveness of OSCC [182,183].

### Infrared spectroscopy

This distinguishes different biomolecules by probing chemical bond vibrations and using these molecular and submolecular patterns to define and differentiate pathological from normal tissues [184,185]. Optical Biopsy technologies have been summarized in (Table 6).

**Table 6:** **Optical biopsy**

**Optical biopsy**	**Technology**	**Light source**	**Information provided**	**Sensitivity %**	**Specificity %**
Auto-fluorescence spectroscopy	Fluorochromes fluorescence (NAD, FADH)	ultraviolet and visible spectrum light	Distinguish malignant tissue by concentration of (NAD, FADH), re-emit green light	81	100
Enhanced dye fluorescence	Fluorochromes fluorescence (protoporphyrin IX)	ultraviolet and visible spectrum light	Distinguish malignant tissue by high concentration of (protoporphyrin IX), re-emit red light	100	100
Ratio imaging	fluorescence (protoporphyrin IX, NAD, FADH)	ultraviolet and visible blue light	Compare a ratio of red emission of (protoporphyrin IX) from malignant cells with the green emission from normal	from 60 to 97	from 75 to 99%
Raman spectroscopy	Raman vibrational spectroscopy	laser-based spectroscopic technique	enabling chemical characterization	80.5	86.2
Elastic scattering spectroscopy	Elastic scattering (white light reflectance)	pulsed xenon arc lamp	provides optical geometrical information	92	79
Differential path-length spectroscopy	Elastic scattering (white light reflectance)	tungsten-halogen lamp	cell biochemistry, intracellular morphology and microvascular properties such as oxygen saturation and average vessel diameter	69	85
Optical Coherence Tomography	scattered light (Fourier domain mode lock swept source-based) OCT	laser-based	Provide provide high-speed three-dimensional OCT pictures	Subjective image required interpretation	Subjective image required interpretation
Angle-resolved low coherence interferometry (A/LCI)	scattered light to measure the average size of different cell structures	laser-based	delivers direct confirmation of precancerous disease to the physician	100	85

## Discussion

Patients with potentially malignant oral lesions referred to specialist centres are faced with long waiting times, leading to significant diagnostic delays [186]. In the United States, the mean time from detection of a potentially malignant lesion by a primary healthcare provider to an appointment with a specialist for evaluation is reportedly 35.9 days [16]. In some cases, this delay exceeds 10 months [15]. In Canada, the diagnosis of oral cancer is delayed by 4.5 weeks, which is significantly shorter than in the United States, where the waiting time is reportedly 18.4 weeks [15-17]. This longer delay is attributed to the disparity in healthcare systems and health insurance-related issues in the United States [15]. Delays in diagnosis of oral cancer by as little as 1 month may contribute to a diagnosis of later stage disease [17]. Moreover, treatment delays of more than 40 days in patients with early-stage oral cancer are associated with an increased risk of locoregional failure, which affects survival [18]. In addition, surgical biopsies are time-consuming, uncomfortable, and stressful for the patient and are relatively expensive procedures. Therefore, development of acceptable noninvasive diagnostic methods that can discriminate benign oral lesions from OSCC and its precursors with minimal false-positive and false-negative results would be beneficial not only for patients but also for society; this would reduce healthcare costs through avoiding unnecessary surgical biopsies and minimise long waiting times for diagnosis at specialist centres [15-18].

TB is one of the oldest noninvasive methods for diagnosing OSCC. Being highly sensitive and moderately specific for malignant lesions, it can be used for screening and to rule out suspicious areas. However, it is an unreliable technique because it is highly subjective and depends on the experience of the investigator. Unlike sampling of uterine cervix cells, analysis of surface epithelial cells of the oral cavity and oropharynx by standard exfoliative cytology (brush biopsy) has proven to be unreliable, identifying as few as 31% of dysplastic tissues [26]. Computerised analysis of oral brush biopsy specimens within the context of premalignant lesions reportedly has a positive predictive value of 58.3%, and with the support of molecular markers including tenascin and cytokeratins, accurate diagnoses are achievable [25].

Exfoliative cytology based on a nanobiochip sensor platform for oral cancer detection has been described. The diagnosis of oral carcinoma by oral brush biopsy with exfoliative cytology based on nanobiochip sensor platform has 97–100% sensitivity and 86% specificity [25,186,187]. Currently, the only commercially available diagnostic adjunct employing exfoliative cytology is the OralCDx® Brush Test with computer-assisted analysis from OralScan Laboratories. In a large multi-centre study, the OralCDx® test demonstrated high sensitivity and specificity (100% and 93%, respectively) for detection of atypical oral epithelia based on morphology, keratinisation, and ploidy patterns [25,187]. The OralCDx® Brush Biopsy (or BrushTest® as it is marketed to dentists) is an in-office test to help ensure that seemingly harmless white or red spots in patient’s mouths are not precancerous or cancerous. OralCDx® has two components: (i) a specially designed brush that is used to painlessly obtain a sample of an oral lesion; and (ii) highly sophisticated computers in a specialty laboratory where specially-trained pathologists analyse the sample and provide a result. In contrast to a typical cytologic smear, such as a Pap smear, which samples cells only from the superficial layer, the OralCDx® brush obtains a complete transepithelial biopsy specimen, collecting cells from all three layers (superficial, intermediate and basal) of the epithelium. OralCDx® requires no anaesthesia and causes no pain and minimal or no bleeding. This technique is very accurate and has been the subject of well-controlled, randomised, clinical trials. In every study in which the same lesion was assessed by both an OralCDx® brush and scalpel biopsy, OralCDx® was shown to be at least as sensitive as scalpel biopsy in identifying dysplasia or cancer. In addition, the positive and negative predictive values of OralCDx® have been repeatedly shown in published studies to be substantially greater than those of other accepted life-saving tests such as the Pap smear, mammogram, and prostate-specific antigen [25,187].

A point of care device for testing saliva to detect oral cancer that is not yet commercially available has been developed by the University of California, Los Angeles (UCLA) Collaborative Oral Fluid Diagnostic Research Laboratory, led by Dr David Wong [188,189]. The test, known as the oral fluid nanosensor test (OFNASET), is a point of care, automated, and easy-to-use integrated system that uses electrochemical detection of salivary proteins and nucleic acids and can measure up to eight different biomarkers in a single test in less than 15 minutes [188,189]. The OFNASET screens for the risk of oral cancer, allowing only test-positive patients to be referred for biopsies [188,189]. It is expected to detect oral cancer at an earlier stage than other techniques, when treatment is more effective and less costly. In addition to detecting oral cancer, the OFNASET will be able to assess for pancreatic, breast, and lung cancers, Sjögren syndrome, Alzheimer disease, and type II diabetes. The developers of this system anticipate obtaining FDA approval for salivary biomarkers of diseases by approximately 2016 to 2017, at which time OFNASET will become commercially available [188,189].

Most optical biopsy technologies are still in experimental studies and clinical trials. The FDA, Health Canada, and the World Health Organization have approved VELscope® (LED Dental) for direct visualisation of autofluorescence of oral cavity and diagnosis of oral carcinoma [190,191]. The VELscope® system now has expanded indications for use based on recently published clinical data in peer-reviewed publications. These indications are as follows [190,191]: (i) to help detect oral cancer and dysplasia; (ii) to help detect mucosal abnormalities that may not be visible or apparent to the naked eye; and (iii) as an aid to identifying diseased tissue around a clinically apparent lesion to help determine the appropriate margin for surgical excision. The VELscope® Vx system is an adjunctive device, which means it must be used together with and as a supplement to traditional intra- and extra-oral head and neck examination. Unlike other adjunctive devices used for oral examinations, the VELscope® Vx does not require any dyes or prolonged testing procedures. In fact, a VELscope® Vx exam can be performed in the dentist’s office during a routine hygiene exam in about 2 minutes [190,191]. In a study of 122 oral mucosa biopsies from 20 patients, Poh et al. showed that VELscope® imaging can identify oral neoplasia in the operating room setting with a sensitivity of 97% and specificity of 94% [99].

A/LCI technology is unique in being able to evaluate microstructure in epithelial tissues up to 500 μm below the surface. The technique uses real-time analysis of light scattered from tissues to detect enlargement of nuclei and other organelle-related changes indicative of early cancer progression. This is an alternative to OCT, which aims to construct a high-resolution image that is analysed either by a trained expert or computer algorithm to provide diagnostic information. In contrast, a/LCI measures diagnostically relevant variables directly and provide them to the physician to enable faster, more direct confirmation of precancerous disease. To date, the technique has been shown to accurately detect pre-cancer in the oesophagus, colon, oral cavity and cervix. Oncoscope (Durham, NC, USA) is now developing a/LCI commercially. In the United States, FDA approval for this application of Oncoscope’s system is expected in 2016 [190,191].

Saliva-based oral cancer diagnosis and optical biopsy are promising noninvasive methods for diagnosing OSCC with high sensitivity and reliable specificity that are easy for primary care practitioners to perform clinically. These technologies provide objective information and do not require special experience for interpretation of the information obtained. They could be widely used in the near future as reliable routine modalities for oral cancer diagnosis and evaluation of the degree of dysplasia of pre-cancerous lesions. It is clearly evident that screening and early detection of cancer and its precursors have the potential to reduce the morbidity and mortality of this disease. These technologies may change the consequences of this disease in the near future.

## Conclusions

It is clear that screening for and early detection of cancer and pre-cancerous lesions have the potential to reduce the morbidity and mortality of this disease. Advances in technologies for saliva-based oral diagnosis and optical biopsy are promising pathways for the future development of more effective noninvasive methods for diagnosing OSCC that are easy to perform clinically in primary care settings.
